# What you can see by developing real-time radioisotope imaging system for plants: from water to element and CO_2_ gas imaging

**DOI:** 10.1007/s10967-018-6324-0

**Published:** 2018-11-10

**Authors:** Tomoko M. Nakanishi

**Affiliations:** Graduate School of Agricultural and Life Sciences, The University of Tokyo, 1-1-1, Yayoi, Bunkyo-Ku, Tokyo, 113-8657 Japan

**Keywords:** Real-time radioisotope imaging system, Water movement, Element movement, Imaging photosynthate movement, Carbon fixation, Plants

## Abstract

Since plants live on inorganic elements, absorbing ions from roots and transferring them to each tissue in a plant is an essential activity. However, little is known about the movement of the elements or water in plant tissue. Though fluorescent imaging is now overwhelmingly used at the microscopic level in biology, especially to visualize chemicals or organelles in a cell, radioisotope imaging has become one of the important methods for human imaging in the medical field. In the case of plant studies, however, real-time radioisotope imaging is little-known among plant researchers. The author has developed radioisotope imaging systems using various radioisotopes to study living plant activity, both for elements and for water. Here we review the real-time radioisotope imaging methods we developed, and show new aspects of plant physiology discovered by live imaging.

## Introduction—Inorganic Elements in Plants [1]

**Fig. 1:**
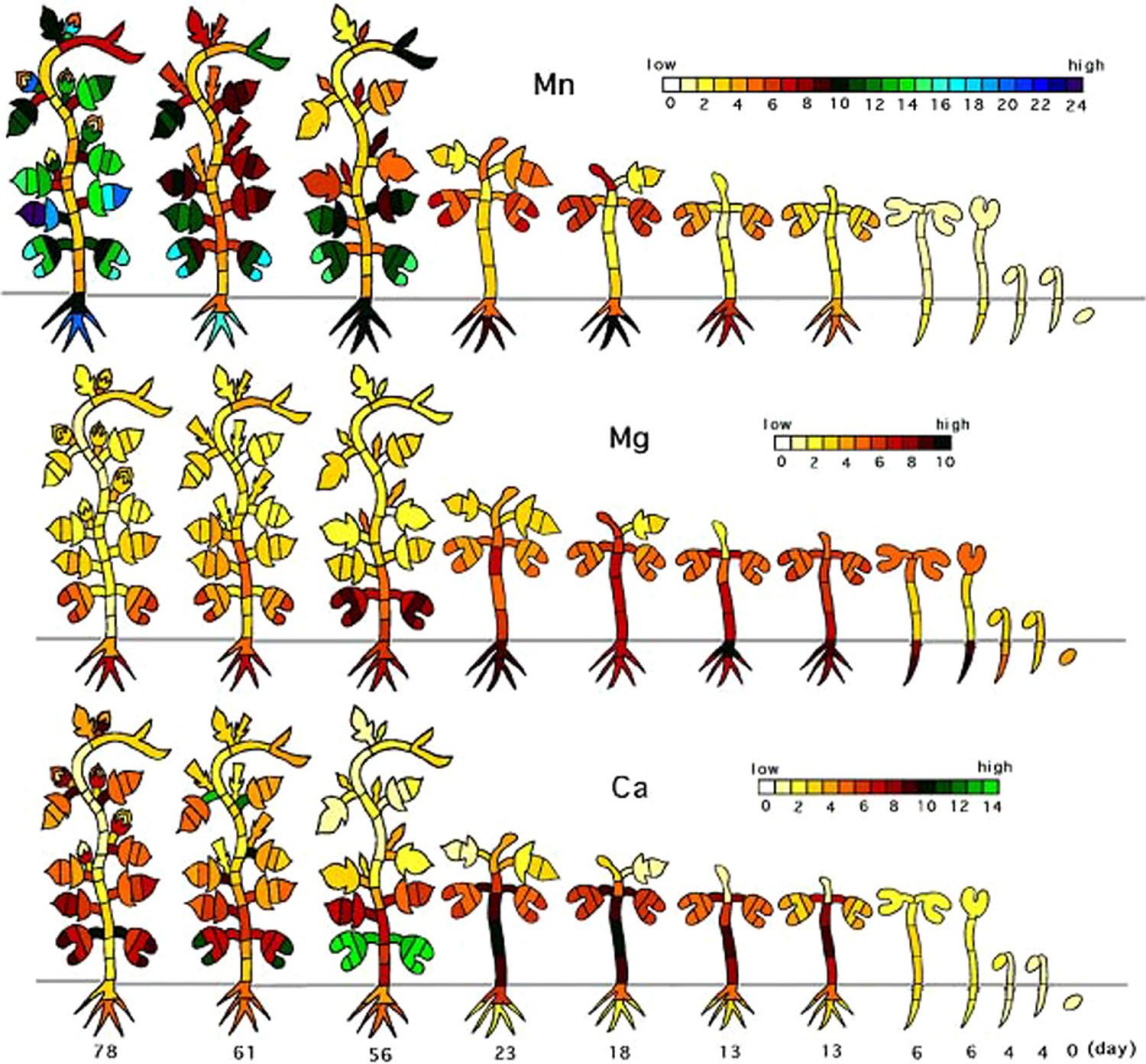
Profile of element concentration in a morning glory during developmental stage [1]. After germination, plant tissue was harvested from day 0 to day 78 and all the tissue was separated. The concentration of the elements in each tissue was measured by neutron activation analysis. Mn, Mg, and Ca profile was selected out of the elements

Plants live on as many as 17 inorganic elements but according to the availability of the elements, they have been developing strategies, such as secreting chemicals or substituting one element with another. Therefore, there are large differences in content of each element among plant species, reflecting different environmental conditions. But what about the concentration difference within plant tissue? Through neutron activation analysis we found that the concentration of each element was drastically different among the tissue of the plant, by factors of 10, 100, 1000, or more. When we analyze the tissue of animals, we do not expect that the concentration of one element in an arm muscle, for example, is 1000 times higher than that in a leg muscle. However, in plants, there is always a concentration gap between different tissues, such as root and above-ground part, internode and leaf stem, for almost all elements. Moreover, there is always an element-specific concentration profile within a plant. For example, heavy elements tend to accumulate in roots. Ca and Mg accumulate during the young age around the cotyledon, and when upper tissue began to develop they moved upward (Fig. 1). Even in one leaf or in one internode, there was a gradient of element concentration. The element profile pattern within a plant was kept in the same manner, throughout the developmental stage. However, in the case of volatile elements such as Cl and Br, some of the elements distributed in a plant were lost from leaves and the content decreased during growth.

## Water Movement [2]

It is not known how the difference in the accumulation pattern specific to each element was occurred. If an element is absorbed as an ion dissolved in water, there must be some relationship between water absorption movement and that of the ion. Therefore, we used ^15^O to label water and measured how water was absorbed and transferred. After supplying the labeled water to the plant we measured the radiation coming from ^15^O to determine how much of the water was moving inside the plant and was transferred to the targeted stem (1 cm of the internode, just above the root). Because of the extremely short half-life of ^15^O (i.e., 2 min), no other research group had attempted to use ^15^O-labeled water to measure the absorption movement in plants. We found that a tremendous amount of water was always leaking out horizontally from xylem tissue in the stem and returning to the xylem again and then moved upward to renew the water at higher position. Since this water circulation in the stem was never reported before, ^3^H-labeled water was used to confirm the water leakage from xylem tissue and returning movement. In this case the imaging could not be the real-time one, for when ^3^H-labeled water was applied and moved inside the plant, the beta-ray energy emitted from ^3^H is too low to detect from outside of the plant. Many plants were prepared, and 5 s after the water was supplied the plant stem was sliced quickly to take an autoradiograph to obtain ^3^H-water distribution.

**Fig. 2:**
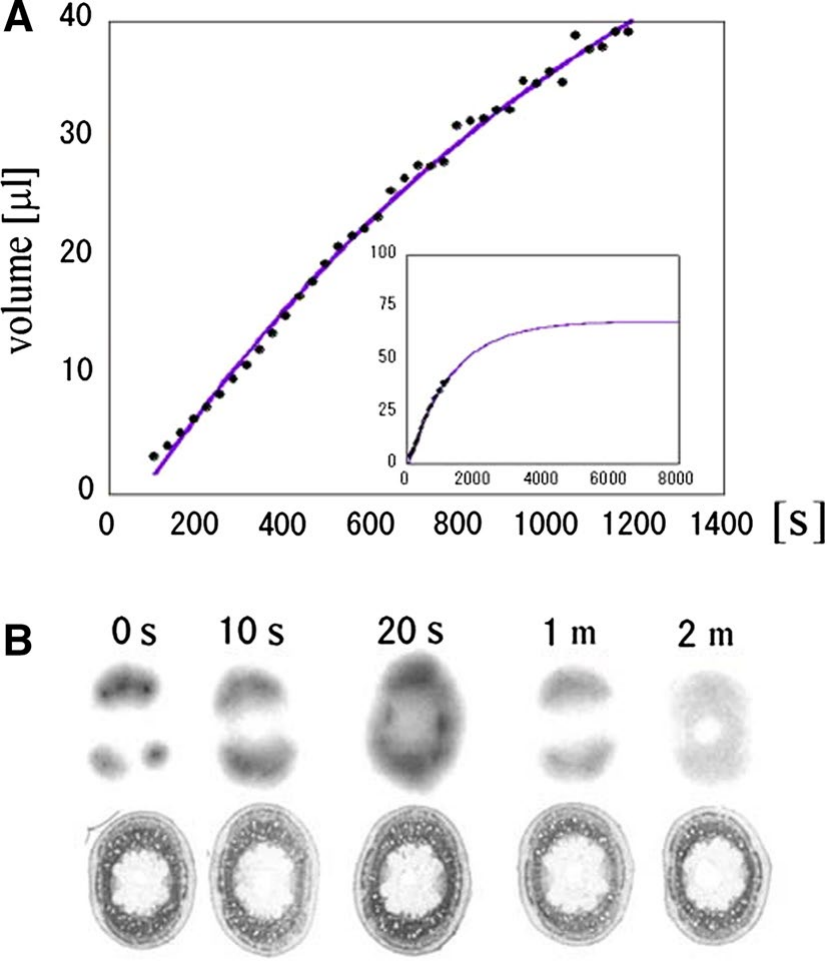
Water absorption of a soybean plant [2]. **a** ^15^O-labeled water was supplied to soybean roots and the amount of water moved up to the 1 cm of the stem in the internode just above the root was measured. Since the half-life of ^15^O is extremely short, 2 min, the absorption measurement was able to be performed until about 1200 s. In spite of the volume of the xylem in the 1 cm stem (2 μL) a large amount of water, close to the volume of the whole 1 cm stem (40 μL), was measured after 1200 s, indicating that a tremendous amount of water was leaking out from xylem. The absorption curve seemed to plateau after about 4000 s through simulation. **b** To confirm the water movement in the stem, ^3^H labeled water was supplied from the root for 5 s and after 10 s to 2 min, the stem was sliced periodically and a water distribution image was obtained by autoradiography. Since the energy of the beta-ray emitted from ^3^H is too low to detect from outside of the plant, real-time imaging is not possible. Therefore, the same experiment was performed using different samples and after some time from application of water, the plant stem was harvested and sliced, therefore all the radiographs were from different samples. The leaked water from xylem tissue was spread out to the whole stem after 20 s and returned to the xylem again and went upward to renew the water already existing there

In the case of ^15^O-water, the water absorption curve was only able to measure until 1200 s after application because of the short half-life of ^15^O. Through simulation it was found that within 20 min about half of the water already existed in the stem was replaced by newly absorbed water (Fig. 2). Water movement we could measure was so different from the movement of dissolved ions that, we wanted to image the real-time element absorption movement.

## Advantage of radioisotope imaging

Radioisotopes have been widely used for tracer work. To get the image, an autoradiography method is employed, where plants were placed on an X-ray film or an imaging plate after application of the radioisotopes to obtain the radioisotope image by radiation exposure. This method provides only a static image, since the plants used for the autoradiography were not able to be used for further experiments.

Because of high sensitivity and the wide range of detection, from trace amount to a large amount of the radioisotopes, autoradiography has come to be used mainly for genetic engineering work; however, this type of the autoradiography has been replaced gradually by fluorescent imaging. Still now, imaging techniques in biology have been quickly developing, especially using fluorescent probes to image chemicals or organic components in cells.

In contrast to fluorescent imaging, there had been no attempt to perform real-time imaging using radioisotopes, except for positron emitters. In the medical field, imaging using positron emitters is well developed and is known as PET (Positron Emission Tomography). In the case of plants, since plants require light, radioisotope imaging has an advantage that it can be conducted either light or dark conditions, whereas fluorescent imaging can be performed only under dark conditions.

PET has become one of the most popular diagnostic tools for patients using the positron emitters with short half-lives, and some research groups have used this kind of imaging technique for plants. However, because of the “positron escape” phenomenon, the intensity of the image can change drastically according to the thickness of the sample, resulting in an image of a stem absent its leaves. The phenomenon also decreases the resolution of the image. As is known, the resolution of the PET images cannot be less than the order of millimeters.

In the case of medical imaging, there are many restrictions for the radioisotopes chosen for human applications: half-lives, radiation energy, etc. However, in the case of plants, to overcome these disadvantages of positron imaging it is preferable to employ other kind of radioisotopes: gamma- or beta-ray emitters, including commercially available ones. The radioisotopes with relatively long half-lives can be the candidates for plants. Since plants require inorganic elements to grow, various kinds of radioisotopes have been used for tracer work to study plant physiology, especially for nutritional work. Therefore, when there is no available radioisotope for the element (for example B, Al, and Si), the study of the element in plant physiology is far behind. Because of this reason, we have been producing ^28^Mg and ^42^K ourselves for imaging and tracer works. ^28^Mg is produced by irradiating an Al target with an alpha beam, followed by chemical separation; and ^42^K is produced from an ^42^Ar generator. The half-lives of both elements are short: 21 h and 12 h for ^28^Mg and ^42^K, respectively.

## Development of Real-Time Radioisotope Imaging System [3–9]

**Fig. 3:**
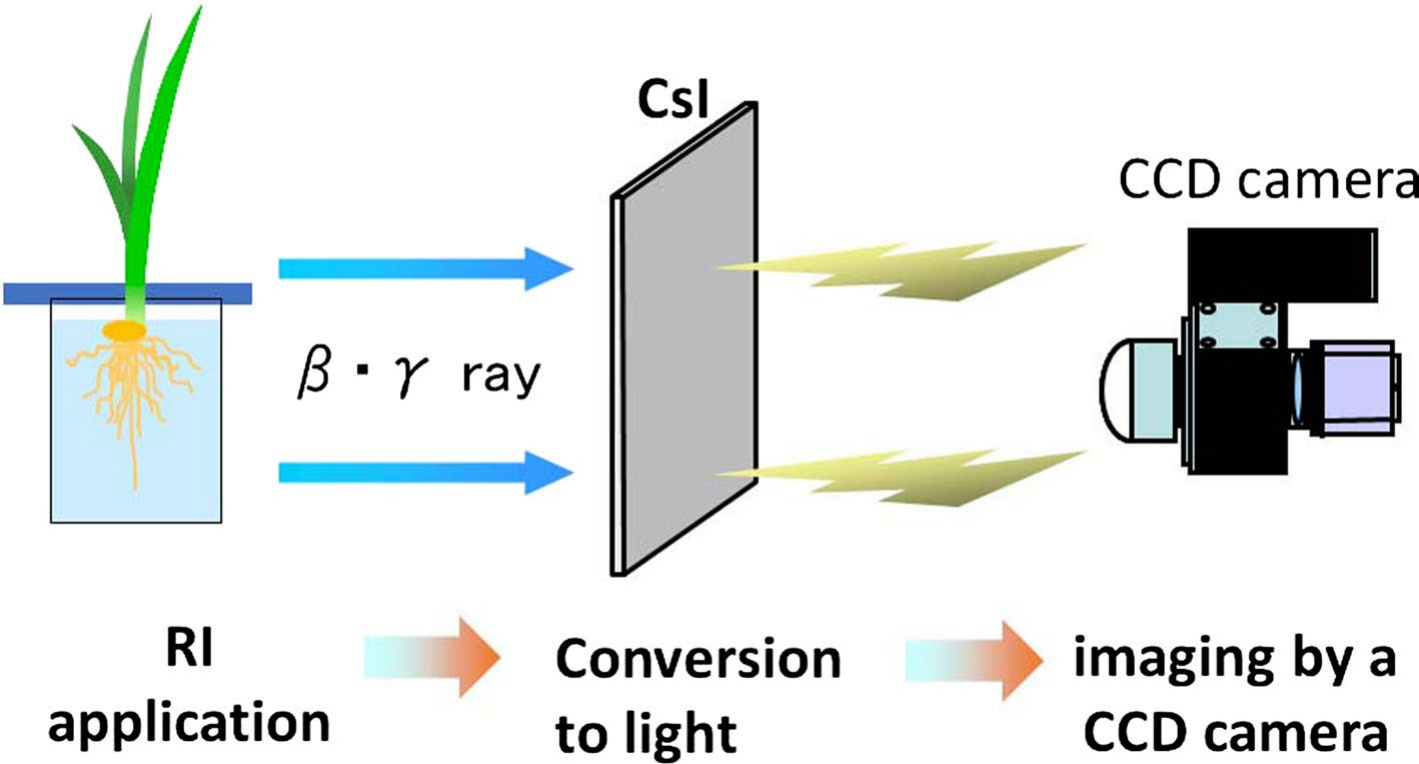
Schematic illustration of real-time imaging system. After application of the radioisotopes to the plant, the radiation of the radioisotopes coming out from the plant was converted to light by a scintillator deposited on FOS (Fiber Optic Plate). Then, the light was introduced to a highly sensitive CCD camera to produce the radiation profile image. Since the light intensity is very weak and the sensitivity of the camera is high, initially, everything was kept in dark to protect the camera. Then, a plant box was prepared where only the above-ground part of the plant was illuminated by light and the plant box was sealed tightly to prevent the leakage of light. Now a chamber is prepared and the light was off when the CCD camera is on. Two imaging systems were prepared; one is for macroscopic and microscopic samples. To image an entire plant, from root to shoot, the macroscopic imaging system is used and for microscopic imaging, a modified fluorescent microscope is used to obtain three images simultaneously, light, fluorescent and radioisotope images

We targeted conventional gamma- or beta-ray emitters and we have been developing the real-time imaging systems for macroscopic and microscopic samples. A schematic illustration of the real-time imaging system is shown in Fig. 3. The radiation emitted from the plant after application of the radioisotopes was converted to light by a scintillator deposited on a FOS (Fiber Optic Plate) and the light image taken by a highly sensitive CCD camera. After evaluation of several types or thickness of the scintillator, now CsI is used. To image an entire plant from root to shoot, the macroscopic imaging system is used, and for microscopic imaging a modified fluorescence microscope is used to obtain three images simultaneously: light, fluorescent, and radioisotope images. We now can image the following 15 elements, ^14^C, ^18^F, ^22^Na, ^28^Mg, ^32^P ^33^P, ^35^S, ^42^K, ^45^Ca, ^48^V, ^54^Mn, ^55^Fe, ^59^Fe, ^65^Zn, ^86^Rb, ^109^Cd, and ^137^Cs.

## Real-Time Radioisotope Images [10]

**Fig. 4:**
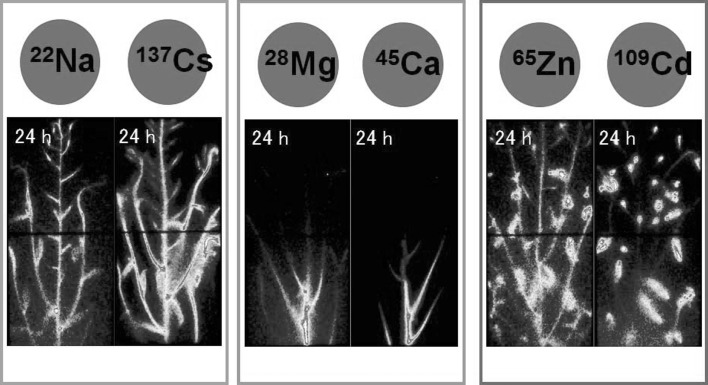
Real-time images of the elements in *Arabidopsis* [10, 13]. The movement of the elements in an *Arabidopsis* plant was visualized. The moving speed is different among the elements. The figure indicates the distribution of some of 6 representative elements after 24 h, which moving speed is different, moderate, slow and very fast. Heavy elements, Zn and Cd move very fast and reach the tip of the leaves after several hours, whereas Mg and Ca movement is very slow, and even after 24 h the distributions of these elements in leaves were not detected

The images obtained to date have shown that the moving speed from root to the highest part of the plant is very element specific. The moving speed of the heavy elements from roots to the tip of the leaves is faster than the other elements, whereas Mg or Ca move very slow (Fig. 4).

**Fig. 5:**
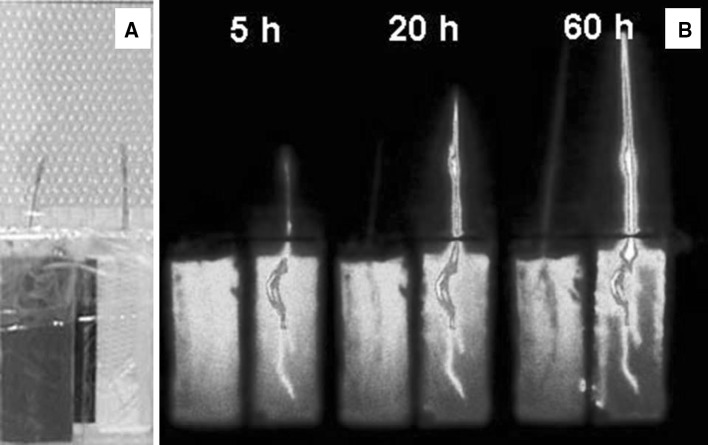
Comparison of ^32^P-phosphate uptake between water and soil culture of a rice plant [7]. The same amount of ^32^P-phosphate was supplied to the rice grown in soil (right) and grown in water culture (left) and the radiation images were taken periodically until 60 h. An integration time per frame was 3 min. In the case of soil culture, since phosphate is liable to be adsorbed in soil, only the limited amount of the phosphate around the root was dissolved and taken up by the roots. Therefore the speed of the growth of the rice plant is much lower in soil culture than that of water culture where ions are dissolved in water and easy to be absorbed by roots

When the plants growing in soil and those in water culture solution were compared, plants grew faster in water culture because of the easier acquisition of elements dissolved in ionic forms in water (Fig. 5), however, the yield of the seeds is low. It seems that the plants growing in water culture do not have the same necessity to produce a large number of seeds to create the next generation, since the nutritional environment is so rich, with plenty of nutrition in water culture. Usually, when the nutritional condition is poor, plants create a large amount of seeds for the next generation to raise the possibility of the survival rate of the species.

The real-time images in Fig. 5 showed that, in the case of phosphate, only a limited amount of the phosphate in soil was absorbed. Since phosphate is liable to be adsorbed in the soil, only the phosphate adsorbed in the vicinity of the root was dissolved by roots for absorption. The images also showed that there was always an accumulation at the root tip where phosphate is required to for proliferation to create new tissues, therefore, sometimes the phosphate moved from upper root to the root tip.

## Visualization of Photosynthesis [11–14]

Another moving force for inorganic ions in a plant is photosynthesis. Although the mechanism of photosynthesis is well studied, there is very little information known about the actual movement of the assimilated carbon compounds/metabolites within plants. Therefore, we prepared ^14^C-labeled carbon dioxide gas and exposed to the plant to it.

**Fig. 6:**
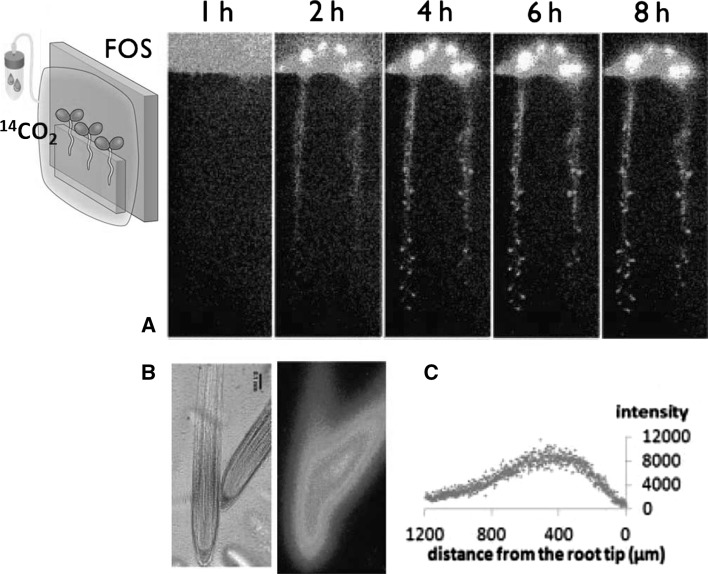
Visualization of how photosynthesis metabolites in leaves are moved to roots [13]. The plants were covered with a polyethylene bag and ^14^CO_2_ gas was supplied for 1 h and the image was taken successively (**a**). The integration time for each image was 15 min and the image was taken at an interval of 1 h. The first image (1 h) shows that the air part was filled with ^14^CO_2_ gas and there is hardly any ^14^C-metabolites coming down. When the photosynthesis proceeds, the metabolites produced from ^14^CO_2_ gas was moved downward to the root tip to create new root tip tissue. **b** When the root tip is imaged under microscopic imaging microscope, ^14^C-metabolites were accumulated in root tips. Bar is 0.1 mm. **c** the distribution of ^14^C-metabolites along the root, obtained from the image analysis

The carbon fixed by photosynthesis creates almost all the tissue of the plants. That means that plants are always creating visible tissue out of invisible carbon dioxide gas in air. It was so impressive to visualize how the carbon dioxide gas in the air was fixed at the above-ground plant and the assimilated carbon metabolites are transferred to create new tissue, for example, new root tip (Fig. 6).

**Fig. 7:**
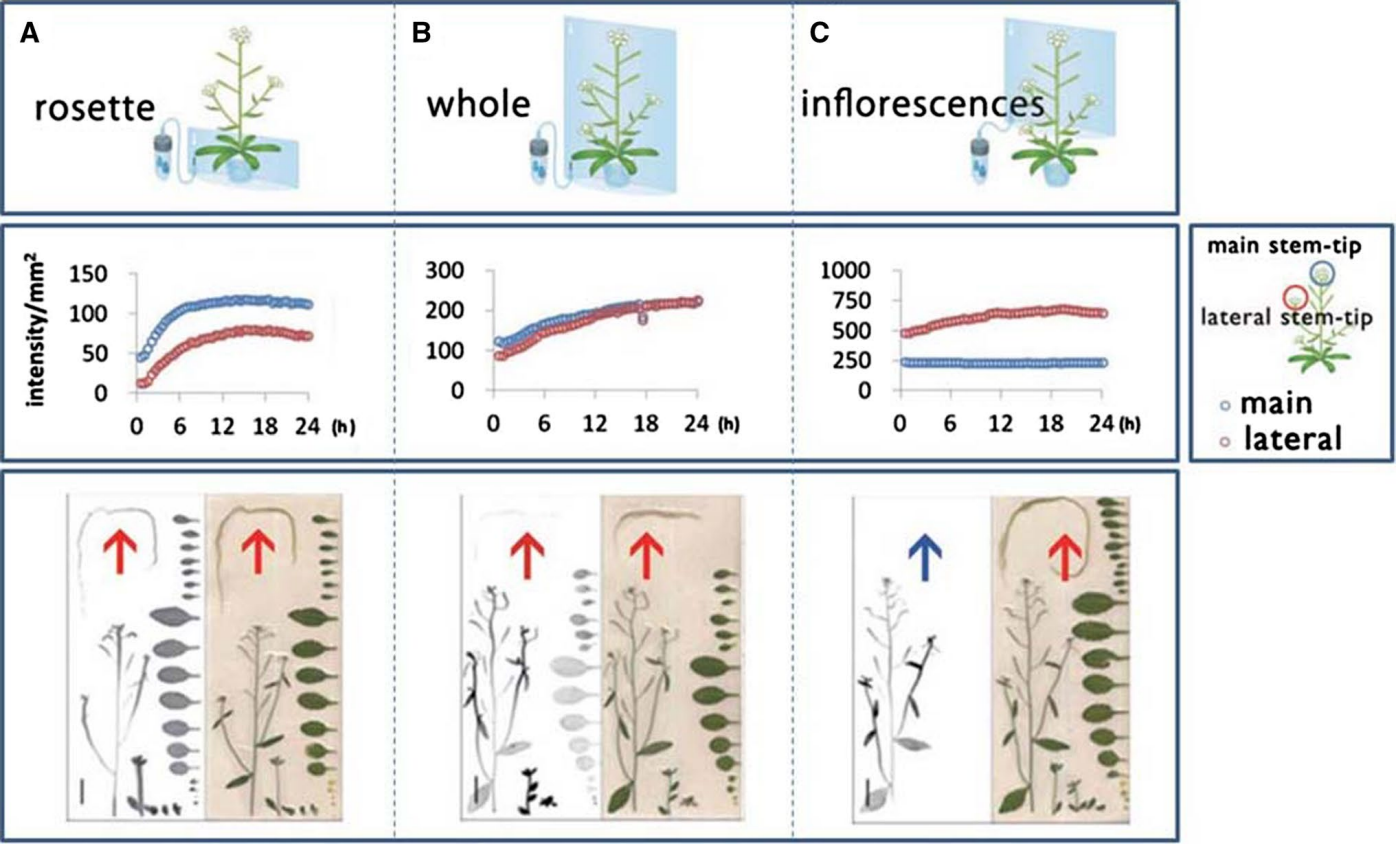
Moving direction of photosynthesis metabolites is different according to the production site [13]. ^14^CO_2_ gas was supplied in three different ways, to rosette, whole shoots and inflorescences for 1 h, to an *Arabidopsis* plant (**a**, **b**, **c**). The integration time for each image was 15 min. Then the amount of ^14^C-metabolites in the main stem tip and lateral stem tip was analyzed to obtain the accumulation curve shown in the middle. The ^14^C-metabolites produced at rosette moved preferentially to the main stem tip, whereas most of the ^14^C-metabolites produced at inflorescences moved to the lateral stem tip. After 48 h, the autoradiograph of the plant was taken to visualize both roots and above-ground part. The lower column shows the autoradiograph (left) and the picture of the sample harvested (right). Only the ^14^C-metabolites produced at inflorescences did not move to the root shown in the autoradiograph in left. The arrows indicate the roots both for autoradiograph and pictures. Bars in autoradiograph are 20 mm

We could show the different direction of the photosynthates movement according to the ^14^C-gas fixation site. In the case of *Arabidopsis*, carbon fixed at the site of the rosette (large leaves spread out in a circle just above the root) is transferred mainly to the main stem tip and roots, whereas the carbon fixed in the small leaves or stems were moved mainly to the tip of a branched stem and does not reach the root (Fig. 7). In the soybean pod, the carbon metabolite from photosynthesis was mainly supplied from the closest leaves to the pod.

## Summary

1. Each element has its specific distribution pattern in a plant
2. Water is circulating in the stem, replacing the water already existing in the tissue with newly absorbed water. In the case of a soybean plant, half of the water already existed in the stem was renewed within about 20 min.
3. A real-time radioisotope system was developed to visualize the movement of each element, from roots to the above-ground part. Ions absorbed move differently compared to water.
4. There was a large difference in element uptake manner and growth of the plant between water culture and soil culture.
5. Carbon fixation and movement of photosynthesis metabolites were able to be visualized. The direction of the photosynthate was different according to the tissue created.
6. In general, elements necessary for plant growth preferentially move to the youngest tissue, including root tips.

It is interesting to note that the carbon metabolites produced from photosynthesis, which constitute the main part of the plant tissue, move differently according the tissue where metabolites were created. To study all these kinetics, utilization of radioisotopes is the best way. From the results of imaging we can discover new physiological aspects of plants.
